# Fracture-Related Infection in Bicolumnar Acetabular Fracture: A Case Report

**DOI:** 10.3390/diagnostics12102476

**Published:** 2022-10-13

**Authors:** Cristiano De Franco, Gabriele Colò, Marco Melato, Alberto Battini, Simone Cambursano, Giuseppe Pietro Logrieco, Giovani Balato, Kristijan Zoccola

**Affiliations:** 1 Orthopaedics and Traumatology Unit, SS Antonio and Biagio and Cesare Arrigo Hospital, 15121 Alessandria, Italy; 2 Orthopaedic Unit, Department of Public Health, Federico II University, 80138 Naples, Italy

**Keywords:** fracture-related infection, 3D printing, acetabular fracture

## Abstract

Case: A 51-year-old man was affected by a fracture-related infection after a bicolumnar acetabular fracture. A significant alteration of the anatomy was present; thus, a 3D-printed model was useful for planning. A two-stage treatment was planned: in the first stage, implant removal with irrigation and debridement was performed, while in the second stage, a new osteosynthesis and implant of a THA were planned. During the second stage, the patient suffered a cardiogenic shock, so a third surgical procedure was necessary to implant THA. Targeted antibiotic therapy was administered eight weeks after the first stage, with the resolution of the infection. Conclusions: The infection was resolved following the recent guidelines and treating it like a periprosthetic infection with a two-stage revision. A collaboration between specialists in orthopaedics and infectious disease, respectively, and using multidisciplinary approach, were mandatory.

## 1. Introduction

Fracture-related infection is a severe complication after a bone injury. Its management is long and complex, with the need for more surgical procedures and collaboration with infectious disease specialists [1,2,3]. A fracture-related infection in the pelvis is more challenging for orthopaedic surgeons than in other districts, due to the pelvis’s anatomy and the fracture’s complexity [4,5]. Bone loss or variation of normal anatomy is often present in this condition. For these reasons, 3D reconstruction and 3D-printed models are becoming increasingly popular and useful among orthopaedic surgeons: 3D printing technology is used for designing patient-specific models, instrumentation, implants, and orthosis, prosthesis and scaffolds [6,7,8]. Using a 3D-printed model can also help an orthopaedic surgeon decide which device to implant, the direction of plates and screws, how to manage the infection, where to debride and how to preserve or restore the function of the involved bone or joint. 

Moreover, important neurovascular structures in close contact with pelvic bone make surgical procedures more dangerous for the patient [9]. Pelvic or acetabular fractures can raise the risk of hip-septic arthritis because of the presence of particular structures in this anatomical region, such as the bladder [10]. Furthermore, the presence of a bone gap with the rupture of the joint capsule can cause a continuity between the hip joint and pelvis. Therefore, an infection by contiguity spread is possible, and due to its proximity to the hip, septic arthritis could occur, so it should be excluded before the last surgical stage [11,12,13,14]. An accurate diagnosis must be obtained to conduct the correct treatment, and the pathogen’s isolation is mandatory to administer suitable antibiotic therapy. Moreover, more surgical stages are needed to perform an accurate septic tissue debridement and allow antibiotics to work correctly [15,16,17]. A multidisciplinary approach between an orthopaedic surgeon and a specialist in infectious disease is needed.

We present a case study of a fracture-related infection of the right acetabulum in polytrauma.

## 2. Case Report

**Figure 1:**
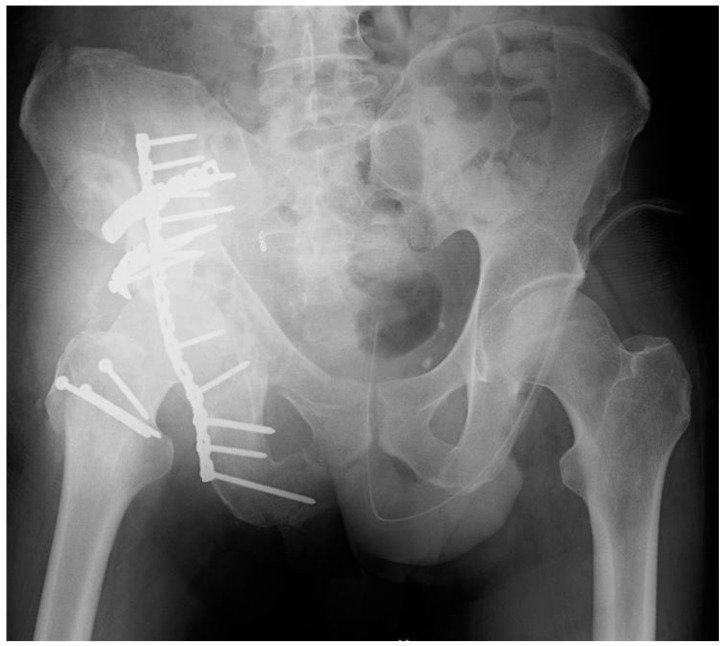
X-ray of the pelvis and hip when the patient came to our attention.

A 51-year-old man was involved in a high-impact trauma (motor vehicle accident) with a right bicolumnar acetabular fracture, according to the Letournel classification. He also had a concomitant left humeral shaft fracture, left pneumothorax, head injury, and liver contusions. He was initially admitted to the intensive care unit of another hospital. When he was stabilized, he was admitted into the orthopaedics department of the same hospital he was admitted. Thus, he was treated with open reduction and internal fixation (ORIF) with plates and screws in both fractures (Figure 1).

**Figure 2:**
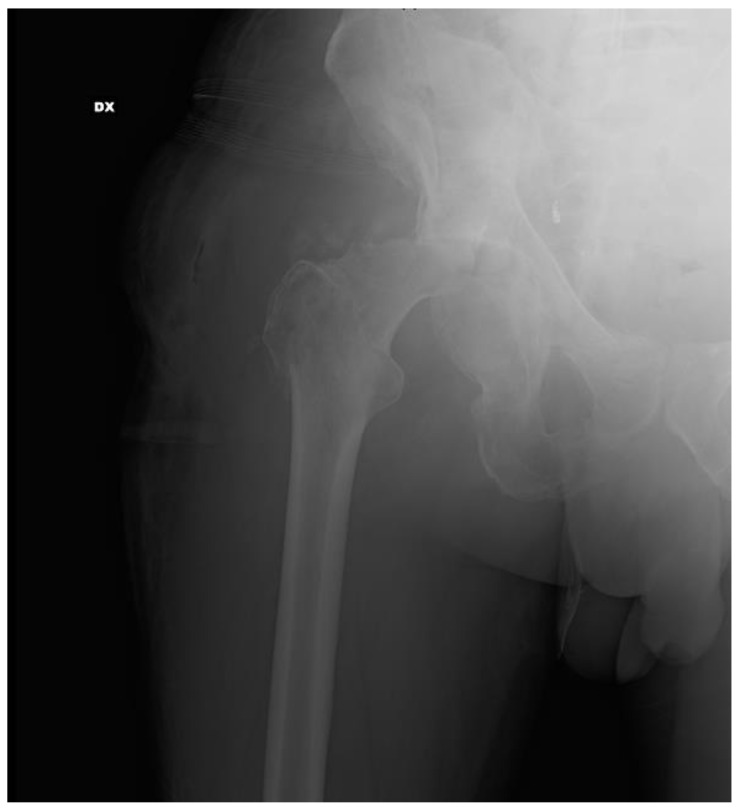
X-ray of the pelvis after the implant removal.

He came to our attention six months after the treatment of the pelvic fracture with ORIF with no weight-bearing, right groin pain, a fever > 38 °C, and a discharging sinus in the right peritrochanteric region next to the surgical scar of the right hip (a KocherºLangenbeck approach was used). The diagnosis of the infection was made thanks to the presence of a discharging sinus. We firstly performed clinical evaluation and blood tests with an ESR (erythrocyte sedimentation rate) and CRP (C-reactive protein) evaluation; then, we decided to perform instrumental planning with X-rays, and computed tomography (CT) scans. We observed a fracture-related infection of the right acetabulum with necrosis of the right femoral head. The patient reported previous oral antibiotic therapy with Amoxicillin (1 g) three times a day and Minocycline (100 mg) two times a day, with the lack of a noted germ. Due to the clinical, instrumental and laboratory indications, we decided to treat him for the fracture-related infection. A two-stage revision was planned using the same approach (Kocher–Langenbeck). In the surgery, we planned to perform implant removal, irrigation, and debridement; in the second surgery, we planned to complete the new osteosynthesis and implant a total hip arthroplasty (THA). In the first stage, we started to perform fistulotomy, then continued with implant removal (plates and screws) from the acetabulum. During the removal of implanted devices, irrigation (about 10–12 L of saline solution) and accurate and aggressive debridement were performed (Figure 2). 

**Figure 3:**
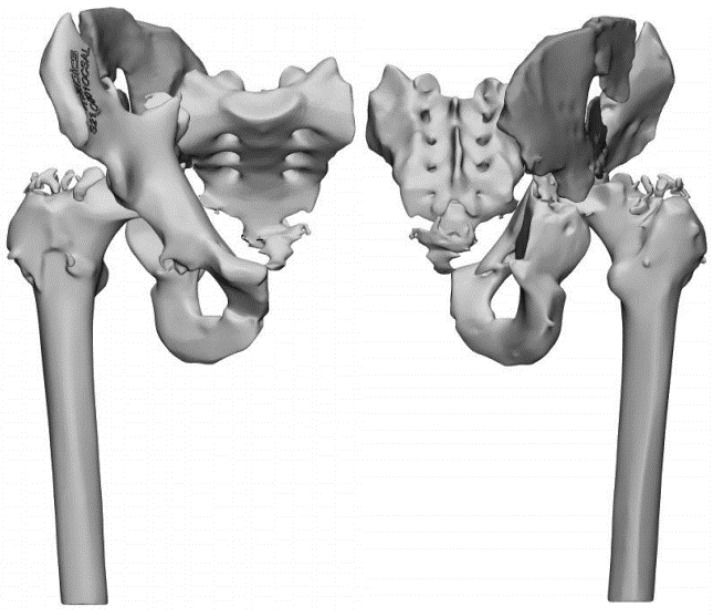
3D reconstruction of the pelvis before the second stage.

Five samples were taken and put in Fluid Thioglycollate Medium; then, cultural examinations were performed by a microbiology laboratory: the presence of Methicillin-Resistant Staphylococcus Aureus (MRSA) was isolated. After the surgical procedure, empiric antibiotic therapy was set up by the specialist in infectious disease: teicoplanin (600 mg) once per day was administered intravenously. After the first dose of teicoplanin, an adverse drug reaction was observed, with rash and severe dyspnea. For this reason, the antibiotic therapy was switched to daptomycin (750 mg) once daily for eight weeks. This antibiotic therapy was confirmed after the results of the cultural examinations were received. In these eight weeks, the negativization of the laboratory examinations (CRP and ESR) was observed with the resolution of the symptoms. The surgical wound healed well, the patient had no fever, and no localized pain was observed. At the suspension of antibiotic therapy, no symptoms were observed, so the second stage was planned. Due to the case’s complexity, accurate planning was performed, with a three-dimensional reconstruction of the pelvis from standard CT scans. We requested a 3D-printed model from an external company (Medics Srl, Turin, Italy) (Figure 3).

Before the second stage, we performed a dry surgery on a 3D-printed model: we selected the type and size of devices for the implant and the direction of implanted components (i.e., the screws). Then, we decided how to reduce the fracture and obtain a new functional acetabulum. In the first stage, we observed significant bone loss and a complete alteration of the normal anatomy. There was a lack of bone in the roof of the acetabulum, severe circumferential osteolysis due to infection, and the interruption of both columns with a bone gap. A standard osteosynthesis could not be performed. In addition, due to the previous infection, the use of an allograft or synthetic graft was contraindicated, so the bone loss could not be treated with it. 

**Figure 4:**
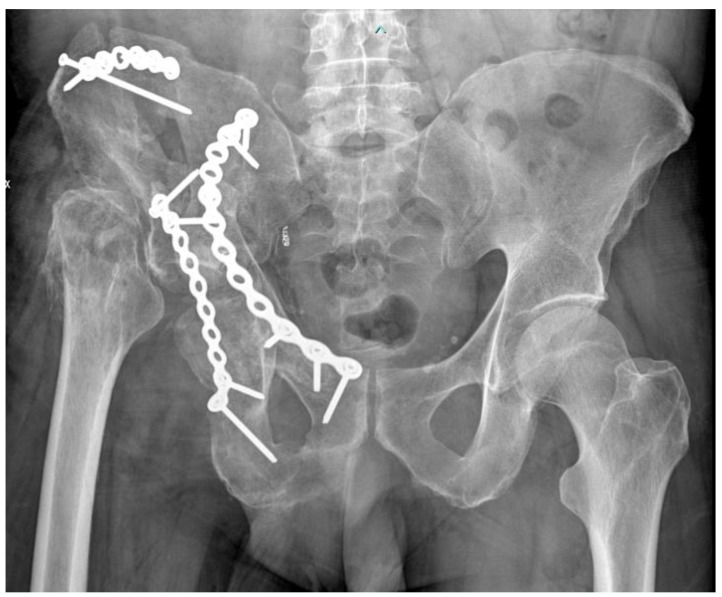
X-ray of the pelvis after the new osteosynthesis of the bicolumnar acetabular fracture.

After three months, the second stage of the surgical procedure was performed. In this stage, two approaches were planned. Firstly, we used a modified Stoppa approach with a lateral window to perform a second debridement, breaking the callus and mobilizing fracture fragments. At this point, thanks to pre-operative planning with a 3D-printed model, we reconstructed the acetabulum and pelvis and, finally, performed the osteosynthesis of the anterior column with two plates and a screw, which was supported by an autologous graft of platelet-rich plasma. Subsequently, we used Kocher–Langenbeck approach, resuming the approaches of previous surgical procedures. From this approach, we conducted a further debridement of the fracture and performed the osteosynthesis of the posterior column (Figure 4). As in the first stage, five samples were taken, and the cultural examinations were negative.

**Figure 5:**
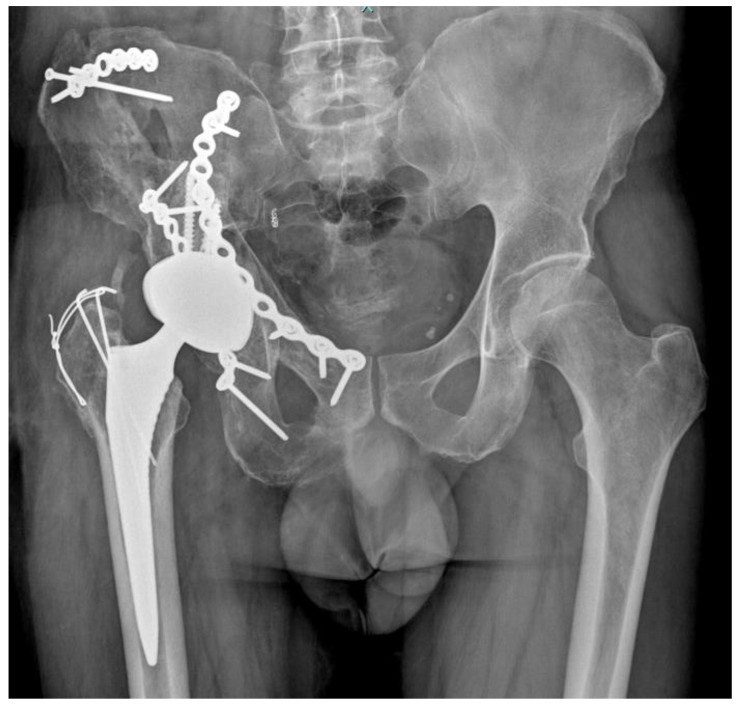
X-ray of the pelvis after the implant of total hip arthroplasty.

Since we had planned to implant a total hip arthroplasty, we conducted the femoral head resection before the new osteosynthesis to perform a better debridement. At this moment, after the osteosynthesis of the posterior column, the patient suffered a cardiogenic shock: the anaesthetist forced the stoppage of the surgical procedure for the safety of the patient. The planned total hip arthroplasty was postponed. After one month, the total hip arthroplasty was performed, resuming the previous Kocher–Langenbeck approach, with no complications (Figure 5).

Postoperatively, the patient had no weight-bearing activity for one month despite the total hip arthroplasty. Prophylaxis for heterotopic calcification was set. The mobilization of the right hip was immediately allowed with no limits. After one month, gradual weight-bearing was allowed with the goal of total weight-bearing at two months. 

One year after the last surgical procedure, the patient had no symptoms, a comparable range of motion of the right hip to the left hip and was fully weight-bearing with no walking aids. An EQ-5D-3L score of 1.000 was observed.

## 3. Discussion

Fracture-related infection of the pelvis can be considered a difficult challenge for orthopaedic surgeons. Many conditions must be regarded for its treatment, and different strategies are involved. The medical team must carefully analyze factors, such as the anatomical region, nonunion of fracture, antibiotic therapy, and type of patient.

The pelvic region is full of pitfalls, and they increase when a fracture-related infection occurs: previous trauma can change the normal anatomy and alter the relationships between anatomical structures of the region; furthermore, the presence of infection makes the discrimination between healthy and pathological tissue very tricky. 

In our case report, the anatomy of the pelvic region was subverted, and there was significant bone loss: it was impossible to restore the original anatomy, so our osteosynthesis aimed to restore the function of the acetabulum. Based on the principle of Y-shape for acetabulum conformation [18], we restored the two columns to fit the acetabular cup. 3D reconstruction and the 3D-printed model played a leading role. According to recent literature, using a 3D-printed model helps orthopaedic surgeons understand the spatial relationships between the fragments of the fracture and how to manage them during the surgical procedure [19].

In terms of bone loss, treatment is very difficult in this case—using synthetic graft or allograft is contraindicated when there is an infection. There is a high risk of not integrating graft: it can become a substrate that promotes the proliferation of pathogens. In our case report, something was needed to stimulate bone healing, and we decided to use an autologous graft without a bone scaffold with the assistance of Platelet-Rich Plasma (PRP) to promote the healing of the fracture, not fill up the gap [20]. There was no strong evidence of its role in bone healing or when it is used without a scaffold. In this situation, mesenchymal stem cells from bone marrow would be recommended; however, it was not possible to withdraw them from the iliac bone due to their proximity to the septic foci [21,22].

The challenge in treating fracture-related infection is also due to the type of treatment: it is a very long treatment, and a multidisciplinary approach is mandatory. For this reason, a good surgeon–patient relationship is desirable. A patient with fracture-related infection becomes discouraged; they experience significant adversity along the way to healing, including trauma, complications after surgery, infection, and a reduction in the quality of life. In this situation, the orthopaedic surgeon has to establish a good connection with the patient: a step-by-step and exhaustive explanation of all possible treatments with their complications is mandatory [23].

When a fracture-related infection is treated, the timing of diagnosis and treatment acquires a relevant role. In the guideline published by Metsemaker et al. [3], the time since osteosynthesis and the onset of symptoms leads us to choose treatment. If the case of an acute fracture-related infection (<2 weeks), irrigation and debridement with implant retention is the best choice [24,25]. In our case, the fracture dated back six or more months ago, with no sign of bone healing. When it occurs, implant removal is the gold standard, which is associated with irrigation and debridement. To irrigate, different solutions are used: saline solution was preferred by the authors, but other solutions can play a relevant role in the future [26]. 

In terms of debridement, removing every possible infection foci and every pathological tissue was necessary: the paprika sign can be useful to understand where and when to stop debridement. If necessary, the orthopaedic surgeon must also remove the relevant functional structure, like muscles and/or bones. After debridement, if the fracture has not healed, a new osteosynthesis is necessary. If possible, an external fixation is preferred, like in the long bone fracture [3]. 

In the literature, there was discussion about the possibility of treating them with a one-stage or a two-stage technique [27]. In our case, it was not possible to apply an external fixator. At the same time, it was impossible to perform a new osteosynthesis at once because of chronic fracture-related infection. Moreover, there was also necrosis of the femoral head, probably secondary to hip septic arthritis. For these reasons, we approached this case with a two-stage revision, like a periprosthetic hip infection. We performed implant removal and irrigation with debridement in the first stage. We decided against the resection arthroplasty in the first stage: a too-aggressive surgical procedure would not have been tolerated by the patient. In the second stage, the patient was involved in an anaesthetic complication (cardiogenic shock). Long surgery times and wide surgical exposure increase the rate of complications: in this case, we had to reduce the risk due to the patient’s conditions [28]. In addition, the resection arthroplasty, according to Girdlestone, increased the instability of the hip joint with more pain and a lack of functionality for the patient; it makes the reconstruction phase more difficult for the orthopaedic surgeon [29]. In the two-stage revision, a hip spacer can be a solution. Its major complication is dislocation due to its intrinsic instability; in our case reports, there was a persistent fracture of the acetabulum, which did not allow the implanting of a hip spacer without significant risk [30,31]. Thus, we treated the fracture-related infection with a two-stage revision, while septic arthritis of the hip with a one-stage revision [14,32]. In the second stage, we planned to perform the definitive osteosynthesis and implant of the total hip arthroplasty after the second aggressive debridement with irrigation [33].

In the treatment of fracture-related infection, a multidisciplinary approach with a collaboration between the orthopaedic surgeon and the specialist in infectious disease is very important: the patient must rely on the approaches of the orthopaedic surgeon and infectious disease at the same time. The orthopaedic surgeon managed the surgical side: implant removal, debridement, osteosynthesis and/or implant of arthroplasty. The specialist in infectious disease manages antibiotic therapy, timing, and adverse drug reactions. It is essential that antibiotic therapy, empirical and then targeted, is set by the specialist in infectious disease, not by the orthopaedic surgeon, based on the story of the patient and as a result of microbiological examinations [3,16,24]. The orthopaedic surgeon can help in the phase of selecting antibiotic therapy: an accurate and appropriate sampling during a surgical procedure (or in pre-operative time, if it is possible) is necessary to eradicate infection. According to Second International Consensus Meeting on Musculoskeletal Infection [34], five samples must be collected during the surgical procedure, using an appropriate instrument and surgical kit. In our case, targeted antibiotic therapy was affected by an adverse drug reaction, so we were obliged to select a second-line antibiotic. The adverse drug reaction of antibiotics can be influenced the treatment negatively, mostly when there is not a second-line antibiotic: when it occurs, a combined consult with the patient is necessary to decide if to continue the antibiotic, treat the adverse drug reaction, or stop it, performing more surgical procedures to mechanically remove the infection.

To our knowledge, this is the first case reported in detail of a chronic fracture-related pelvis infection sustained by MRSA with an adverse drug reaction, where complete healing was achieved with the total hip arthroplasty implant. In addition, we described the relevant role of 3D reconstruction and a 3D-printed model in managing an acetabulum fracture. The use of graft was limited by infection, so we tried to use Platelet-Rich Plasma. The application of principles of treatment of periprosthetic joint infection guided us to the resolution of the infection with excellent results at the last follow-up appointment. This shows that a strong collaboration between the orthopaedic surgeon and specialist in infectious disease was the key to the treatment of this pathology.

## 4. Conclusions

Fracture-related infection with necrosis of the femoral head is a severe complication after a bicolumnar acetabular fracture. This pathology was treated following the recent guidelines and applying the principles of the treatment of periprosthetic joint infection. The treatment of fracture-related infection is very difficult, so every instrument at our disposal can be useful, including the 3D-printed model, the use of a graft, and so on. Furthermore, a strong collaboration between the orthopaedic surgeon and specialist in infectious disease is mandatory to reach the full recovery of the patient.

## Data Availability

Not applicable.
